# Genotyping errors can affect indirect predictions of young selection candidates: A simulation study

**DOI:** 10.3168/jdsc.2025-0882

**Published:** 2026-02-28

**Authors:** Alberto Cesarani, Fernando Bussiman, Jorge Hidalgo, Matias Bermann, Ignacy Misztal, Daniela Lourenco

**Affiliations:** 1 Department of Animal and Dairy Science, University of Georgia, Athens, GA 30602; 2 Dipartimento di Agraria, University of Sassari, Sassari 07100, Italy

## Abstract

This study aimed to evaluate the effect of genotyping errors on indirect predictions (IP). For that, we simulated a dairy cattle population with 5 replicates, using QMSim. Benchmark GEBV for young animals (n = 11,250) were computed using a full single-step genomic BLUP (generations 11–15). Three SNP error rates (5%, 10%, and 20%) were applied to the genotypes of selection candidates (generation 15). A reduced analysis with generations 11 to 14 (44,500 animals) was conducted to obtain SNP solutions for computing IP in young animals using either the correct genotype files or files with genotyping errors. The average IP values decreased as the error rate increased. The correlation between the benchmark GEBV and the IP obtained with the correct genotype was 0.98 ± 0.01. As expected, this correlation decreased with increasing genotyping errors, reaching its lowest value (0.93) at 20% errors. The regression coefficient of GEBV on IP increased (i.e., more underdispersed breeding values) as the error rate increased: b_1_ moved away from 1.01 ± 0.02 (no errors) to 1.33 ± 0.03 (20% errors). Results of this study highlight the importance of accurate genotype calls to achieve precise and unbiased IP when performing IP for young animals with genotypes.

## Highlights

• Breeding values for young, genotyped animals and no phenotypes can be estimated via indirect predictions.
• Indirect predictions depend only on SNP contents and effects.
• Indirect predictions decreased as the error rate increased.
• Correct genotypes for candidates are needed to obtain reliable indirect predictions.

## Graphical Abstract

**Figure:**
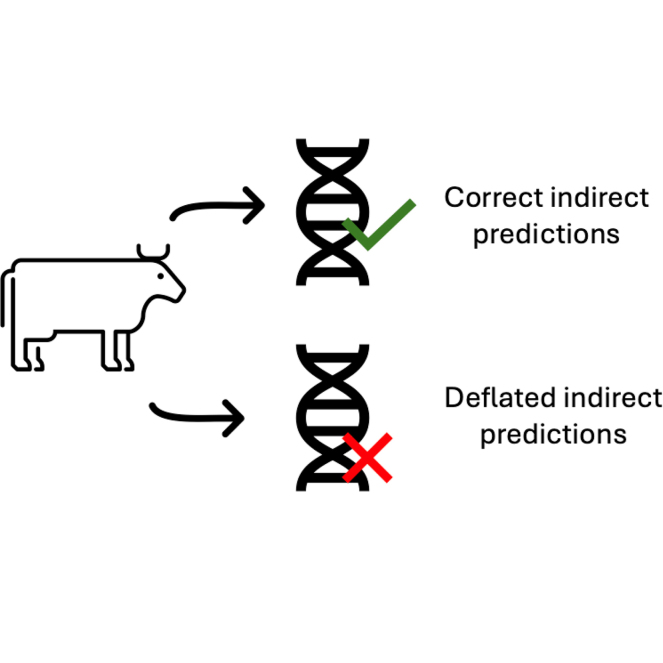
**Summary:** When young animals with genotypes have no phenotypes or progeny, their breeding values can be estimated in a second step via indirect predictions. The latter depend on single nucleotide polymorphism (SNP) contents and SNP effects estimated using the single-step genomic best linear unbiased predictor model. This study investigated the effect of genotyping errors on the indirect predictions of young candidates. The average indirect prediction values decreased as the error rate increased and became more distant from the benchmark genomic estimated breeding values. Genotypes with low error rates are needed for reliable indirect genomic predictions.

Genomic selection (**GS**) uses SNP markers to estimate the genomic merit of individuals. This technology enabled breeders to reduce the generational interval while enhancing selection accuracy and increasing response to selection. Using genomic information has led to significant improvements in both productive and functional traits, with particular emphasis on the latter, for which measurements are often costly or difficult to obtain, and heritability is frequently low (Calus et al., 2008). Among the various models used in GS, the single-step genomic BLUP (**ssGBLUP**; Legarra et al., 2009; Christensen and Lund, 2010) has become the most widely used worldwide (Bermann et al., 2022).

The ssGBLUP model combines phenotypic, pedigree, and genomic information into a single framework, enabling GS for the majority of livestock populations, when only a portion of the animals are genotyped. The ssGBLUP involves the inverse of a realized relationship matrix (**H**^−1^; Aguilar et al., 2010) that contains the inverses of the pedigree-based (**A**^−1^) and genomic (**G**^−1^) matrices. One of the early challenges of ssGBLUP was that inverting **G** was possible only for up to about 150k genotyped animals (Fragomeni et al., 2015). However, some populations now have millions of genotyped animals (Cesarani et al., 2022; Vandenplas et al., 2023). Inverting the genomic relationship matrix with this large number of genotypes required very efficient algorithms, such as the algorithm of proven and young (**APY**; Misztal et al., 2014a; Misztal, 2016). In APY, genotyped animals are divided into core and noncore, and only the portion of **G** with the core animals is directly inverted.

Other approaches to reduce the computational burden of large-scale genomic evaluations include removing genotyped animals that have already been culled (Bussiman et al., 2023) or using indirect predictions (**IP**) for young candidates (Lourenco et al., 2015; Lourenco et al., 2018; Garcia et al., 2020). When young genotyped animals do not have their own or progeny phenotypes, they do not contribute phenotypic information to the system (Bussiman et al., 2023) and, thus, their inclusion in the main genomic analysis may not be worthwhile. The breeding value of these young candidate animals can be predicted as a linear function of SNP effects back-solved from the GEBV of genotyped animals included in the ssGBLUP evaluations.

Utilizing IP for young candidates offers advantages, including reduced computing time, enabling weekly or even daily evaluations. Lourenco et al. (2018) computed IP for the American Angus population and demonstrated the feasibility of this technique when SNP effects are obtained from ssGBLUP GEBV. In the same breed, Garcia et al. (2020) used APY to investigate the accuracy of IP with a larger number of genotyped animals. These authors reported that at least 15,000 core animals are needed to represent the complete genotyped population (Pocrnic et al., 2016) and to obtain trustworthy predictions of SNP effects. Tsuruta et al. (2021) tested the application of IP using 18 linear type traits in US Holsteins by calculating SNP effects using randomly selected genotyped animals ranging from 15k to 60k. These authors concluded that SNP effects computed using 25k to 35k random genotyped animals can lead to accurate and unbiased IP.

All these studies agreed that IP represents a valid approach to drastically reduce computing time (Wiggans et al., 2015) and to avoid potential biases introduced by using young genotyped animals (Bradford et al., 2017, 2019). Because of the benefits, IP are becoming an integral part of the genomic evaluation pipelines for large, genotyped populations. Previous studies on IP have considered genotypes from real populations and focused on the agreement between GEBV and IP, without accounting for errors arising from incorrect SNP content. Most livestock populations rely on genotype imputation because of the wide range of SNP chips available, and it is well known that imputation can introduce errors (Pook et al., 2020). Genotyping errors might have a detrimental effect on the quality of IP, which is important to study, as GEBV may be less sensitive to genotyping errors because it also accounts for other sources as parent average, progeny contribution, and yield deviation (Lourenco et al., 2015). To assess the impact of genotyping errors in IP when SNP effects are back-solved from GEBV from ssGBLUP evaluations, we used simulated data.

The data used in this study were simulated using QMSim v2.0 (Sargolzaei and Schenkel, 2009). First, a historical population (with no selection, no migration, random mating, an equal sex ratio, and independent generations) was created, starting with a population of 1,000 animals and decreasing to 100 animals. Two bottleneck effects were included in the historical population: First, the population size was reduced from 1,000 to 200 for over 1,020 generations, and second, from 200 to 100 over 1,000 generations. This was done to ensure an effective population size (***N_e_***) of 100 and that not all markers and QTL were fixed. To expand the resulting population, the animals from the last historical generation (50 males and 50 females) were randomly mated (by random union of gametes) for an additional 8 generations without selection, assuming an exponential growth of the number of dams. After that, 25 males and 2,500 females (*N_e_* = 100; Wright, 1931) were randomly selected to form the current population (i.e., the population used in the analyses). A total of 15 generations were simulated in the current population (with a 30% growth of the breeding population) and sire and dam replacement ratios constant at 60% and 20%, respectively. An assortative mating design, along with selection for higher predicted breeding values (EBV) and culling the lowest EBV, was used to introduce selection. The heritability of the trait was set to 25%, from which 5% was due to the residual polygenic effect. To mimic the real cattle genome, we simulated 29 chromosomes with lengths ranging from 40 to 146 cM (totaling 23.33 M), and varying numbers of SNPs (from 1,158 to 4,367) and QTL (from 4 to 70). Recurrent mutation rate of SNP and QTL was assumed to be 1 × 10^−4^. The final total numbers of SNPs and QTL were 70,000 and 1,000, respectively. Phenotypes were recorded on both males and females. Generations 11 to 14 were considered as the training dataset (44,500 animals), whereas the last generation (12,250 animals) was used as the validation dataset.

The simulation was replicated 5 times to obtain standard errors for the results. A ssGBLUP model was run using the pedigrees, phenotypes, and genotypes of both the training and validation datasets (i.e., including young candidates) to estimate the benchmark GEBV using BLUP90IOD3 (Lourenco et al., 2022). Then, the last generation was removed from the analysis and a reduced ssGBLUP was carried out considering generations 11 to 14; GEBV of the training population were back-solved using postGSf90 (Misztal et al., 2014b) to obtain SNP effects $\left(\hat{a}\right)$ as follows:

[1] $$\begin{array}{c} \hat{a}=E\left(a|{\hat{u}}_{t}\right)=\left(1-\alpha \right)d\frac{1}{2\sum_{l=1}^{n}p_{l}\left(1-p_{l}\right)}Z_{t}^{'}G_{tt}^{*-1}{\hat{u}}_{t},\; \end{array}$$

where **a** is the vector of SNP effects, ${\hat{u}}_{t}$ is the vector of GEBV for animals in the training (*t*) set (generations 11 to 14); *p_l_* is the *l*th allele frequency; **Z***_t_* is the centered gene content matrix for the animals in the training set; $G_{tt}^{*-1}$ is the inverse of the genomic relationship matrix for the animals in the training set after tuning and blending (*), computed as $G_{tt}^{*}=\left(1-\alpha \right)\left(11^{'}c+dG_{tt}\right)+\alpha A_{22_{tt}},$ where $A_{22_{tt}}$ is the pedigree relationship matrix for genotyped animals in the training set, *α* = 0.05 is the blending parameter; and tuning parameters *c* and *d* were computed as in Vitezica et al. (2011): $d=1-\frac{1}{2}c,$ with $c=\bar{A_{22_{tt}}}-\bar{G_{tt}}$ (i.e., the difference of means). The IP were obtained as $\mathrm{IP}=Z_{v}\hat{a},$ where **Z***_v_* is the centered gene content matrix for the animals in the validation (*v*) set (i.e., generation 15). Four different scenarios were tested, differing on the level of error in **Z***_v_*: (1) correct, where the genotypes of selection candidates did not contain errors; (2) **E05**, where 5% of the SNPs (3,500 markers) of selection candidates were randomly changed; (3) **E10**, where 10% of the SNPs (7,000 markers) were randomly changed; and (4) **E20**, where 20% of the SNPs (14,000 markers) were randomly changed. The changes were such that SNPs coded as 0 could receive 1 or 2, SNPs coded as 1 could receive 0 or 2, and SNPs coded as 2 could receive 0 or 1.

To evaluate the impact of genotyping errors, correlations between the benchmark GEBV (obtained using data on selection candidates using full ssGBLUP with correct genotypes) and the 4 different IP sets were computed. Root mean square error (**RMSE**), mean standardized bias (average IP − average GEBV divided by the square root of the additive genetic variance), and variance ratios between GEBV and IP were also computed. Moreover, the benchmark GEBV were regressed on the IP to evaluate intercepts (expected to be 0) and regression coefficients (expected to be 1). All results were expressed as mean ± SE across the 5 simulated replicates.

Boxplots of the benchmark GEBV and the IP computed using different genotype files for candidates are in Figure 1. The average values (± SE) across replicates were 1.14 ± 0.01 for GEBV, 1.17 ± 0.01 for IP correct, 0.94 ± 0.01 for IP E05, 0.71 ± 0.01 for IP E10, and 0.26 ± 0.01 for IP E20. The breeding values for selection candidates decreased as genotyping errors increased, indicating that genotyping errors underestimate IP. Table 1 shows correlations (Pearson and Spearman) between benchmark GEBV and IP across all scenarios. As expected, the largest correlations (0.98 for Pearson and 0.97 for Spearman) were found when IP were obtained using the correct genotype file. Correlations decreased as the percentage of errors increased; the lowest values were observed for E20, with Pearson and Spearman correlations of 0.93 and 0.92, respectively. Standard errors across the 5 replicates were 0.01 for both correlations.

**Figure 1:**
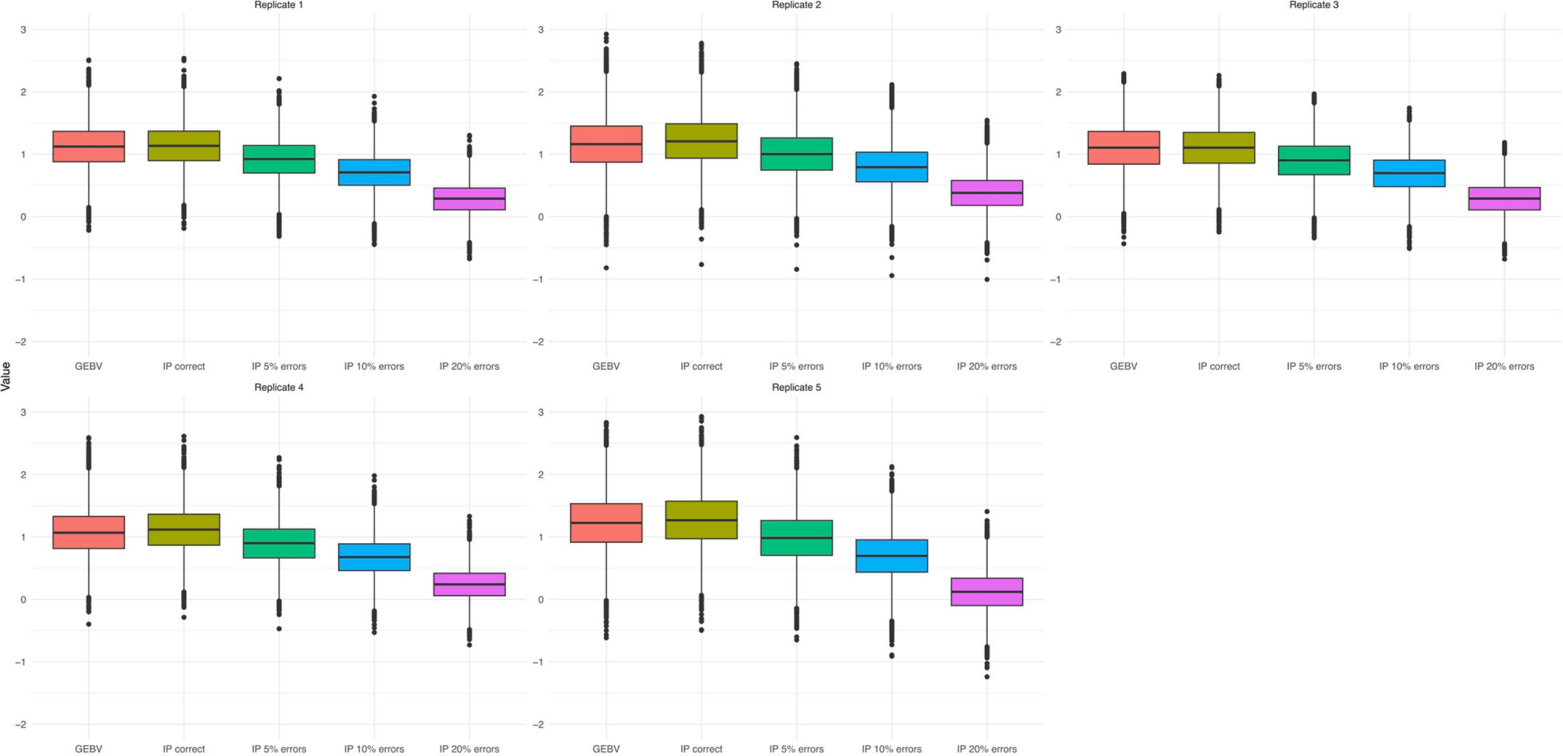
Boxplot of benchmark GEBV and genomic indirect predictions (IP) computed using different genotype files for young candidates. The box represents the interquartile range (IQR). The line drawn inside the box indicates the median. The whiskers were computed as 1.5 × IQR. The dots represent outliers (observations outside the range of the whiskers).

**Table 1:** Correlations (±SE across 5 replicates) between benchmark GEBV and genomic indirect predictions (IP) computed using different external genotype files (correct and with 5%, 10%, or 20% of errors) and results of the regression of GEBV on IP^1^

File	Correlation GEBV, IP		Regression GEBV ~ IP	
	Pearson	Spearman	b_0_	b_1_
Correct	0.98 ± 0.01 (0.98, 0.98)	0.97 ± 0.01 (0.96, 0.97)	−0.04 ± 0.02 (−0.04, −0.03)	1.01 ± 0.01 (1.00, 1.01)
E05	0.96 ± 0.01 (0.96, 0.96)	0.96 ± 0.01 (0.96, 0.96)	0.13 ± 0.02 (0.12, 0.13)	1.07 ± 0.01 (1.07, 1.08)
E10	0.95 ± 0.01 (0.95, 0.96)	0.95 ± 0.01 (0.95, 0.95)	0.32 ± 0.03 (0.31, 0.32)	1.15 ± 0.01 (1.14, 1.15)
E20	0.93 ± 0.01 (0.93, 0.93)	0.92 ± 0.01 (0.92, 0.93)	0.79 ± 0.07 (0.78, 0.79)	1.33 ± 0.01 (1.32, 1.34)

1 Values within parentheses represent the average of 95% CI across the 5 replicates. All values were significantly different from zero (*P* < 0.0001). b_0_ is the intercept; b_1_ is the regression coefficient.

Similar results were found when benchmark GEBV were regressed on IP (Table 1): The values of intercept and regression coefficients increased as the error rate increased. The regression of benchmark GEBV on IP using the correct genotype file yielded desirable values: an intercept close to 0 and a regression coefficient close to 1. These values suggest that IP obtained with the correct genotypes shows a similar distribution and are therefore good estimators of GEBV. The introduction of errors led to underestimated IP (i.e., smaller values than the benchmark GEBV). The regression coefficient in the E20 scenario was 32% larger than the value observed using the correct file (1.33 vs. 1.01). The results for the intercept were even worse. The intercept for E20 was 20 times larger than the value observed in the correct scenario (0.79 vs. −0.04; Table 1). These results reflected a strong reduction in average IP, from 1.17 to 0.26 (Figure 1). The same results, both in terms of correlations and regression statistics, were found using the APY to obtain the SNP solutions (data not shown). Table 2 shows the RMSE, the mean standardized bias, and the variance ratio between benchmark GEBV and the different sets of IP. As expected from the results reported in Table 1, the RMSE increased with increasing error rate, whereas the mean bias (the difference between the IP and GEBV) became more negative because the average IP decreased with higher errors in the validation population (i.e., E05, E10, and E20). Finally, the variance ratio (the ratio between the variance of GEBV and the variance of IP) also increased as the error rate increased.

**Table 2:** Root mean square error (RMSE), mean standardized bias, and variance ratios between GEBV and genomic indirect predictions (IP) computed using different external genotype files (correct and with 5%, 10%, or 20% of errors)^1^

File	RMSE (GEBV, IP)	Bias (IP − GEBV)	Variance ratio (GEBV/IP)
Correct	0.10 ± 0.00	0.06 ± 0.02	1.08 ± 0.01
E05	0.23 ± 0.01	−0.41 ± 0.03	1.25 ± 0.01
E10	0.44 ± 0.03	−0.88 ± 0.06	1.45 ± 0.02
E20	0.89 ± 0.06	−1.83 ± 0.12	2.04 ± 0.02

1 All values are expressed as mean ± SE across the 5 replicates.

In the linear regression model, a fundamental assumption is that values of the predictor variables are known without error. On the contrary, when predictor variables contain errors, the uncertainty will dilute the regression relationship (attenuation bias), increasing the background variation (Bulmer, 1979). Because of IP underestimation due to genotype errors in the validation population, the regression of benchmark GEBV on IP showed an increasing slope and intercept (Table 1). Genotyping errors decrease the accuracy of predictions and, therefore, slow the genetic progress in the population. To verify the impact of noisy genotypes on the estimation of SNP effects, we assumed a 20% error in the genotypes of the training population, and considered correct genotypes for the validation population. Whereas the average IP with both correct training and validation genotypes was 1.17, it was 1.51 with 20% error in the training genotypes and correct genotypes for the validation population (data not shown).

In routine evaluations, genotypes are usually imputed to a common SNP panel. Weigel et al. (2010) investigated the predictive ability of IP computed using imputed genotypes in Jersey cattle. These authors found that IP accuracy may be limited when there are fewer than 1,500 SNPs. However, Wang et al. (2016) reported a minimal impact of imputation errors on the GEBV for residual feed intake in beef cattle. In genomic predictions, phenotypes, pedigree, and genomic data are used to estimate GEBV, whereas IP rely solely on SNP effects and gene contents.

A negative effect of marker genotyping error on the accuracy of genomic evaluations has already been reported in the literature by Akbarpour et al. (2021). In a simulation study, these authors found the lowest correlation between estimated and true breeding values with 20% of marker genotyping error (i.e., like in the present study). Moreover, unbiased regression coefficients were reported when GEBV were obtained without genotyping errors. Khatkar et al. (2012) reported correlations between daughter trait deviations and IP using imputed or actual genotypes. These authors reported negligible loss in accuracy when using the actual 50k SNPs or genotypes imputed from 3k or 7k panels. However, there are substantial differences between their findings and those of the present study. A possible explanation is that Khatkar et al. (2012) had a low imputation error rate and estimated SNP effects using a different model that treated daughter trait deviations as phenotypes, resulting in low accuracies (all <0.56; Khatkar et al., 2012). In the present study, SNP effects were estimated using ssGBLUP, and higher correlations (compared with Khatkar et al., 2012) were observed between GEBV and IP. Several studies reported large correlations between GEBV from ssGBLUP and IP (Lourenco et al., 2015; Garcia et al., 2020, 2022; Vandenplas et al., 2023). Garcia et al. (2020) investigated the impact of IP on birth weight, weaning weight, and postweaning gain in American Angus cattle. They examined the effects of different core definitions in the APY algorithm on IP. These authors found the lowest correlation (0.96) when IP for more recent animals (born in 2015) were computed based on SNP effects estimated from older animals (born in 2013) as core. As already mentioned, SNP effects to be used for IP should be estimated from a sample of genotyped animals that accurately reflects the genomic variability of the population (Hidalgo et al., 2023). This was confirmed by Garcia et al. (2022), who demonstrated that the number of genotypes used to estimate SNP effects influences the correlation between GEBV and IP: correlations increased as the number of genotypes used for back-solving SNP effects increased. Correlations were 0.89, 0.97, and 0.99, respectively, when 2k, 5k, and 10k genotyped animals were considered in the model for estimating SNP effects. As mentioned previously, our tests (data not shown) suggest that genotyping errors should have similar impacts on the accuracy of IP predictions obtained using or not using a sparse representation of **G**^−1^ (i.e., APY).

The IP derivation in our study is a robust approximation of GEBV. It is important to note that in this derivation, we ignored the residual polygenic effect, which has a marginal effect because the assumed proportion was 5%; however, larger residual polygenic effects have a more significant contribution. In such cases, it is important to consider the exact IP derivation by Liu et al. (2016), recently investigated by Strandén et al. (2022) and Vandenplas et al. (2023). Strandén et al. (2022) demonstrated that accounting for a residual polygenic effect (20% in their study) in the derivation of the IP increased the GEBV-IP correlation from 0.966 to 0.996 for calving difficulty data in dairy cattle. Vandenplas et al. (2023) revisited and extended the IP formula by incorporating a covariate that modeled the difference between pedigree and genomic bases. Using the same calving difficulty data and a multitrait model with a residual polygenic effect of 20%, they found that an IP based solely on the direct genomic value had an average correlation with GEBV of 0.948 or higher, whereas their most accurate method achieved an average correlation of 0.996 across traits.

In the present work, we investigated the impact of 3 different genotyping errors on the accuracy and bias of IP for genotyped young selection candidates. Overall, our results demonstrate that genotyping errors systematically reduce the accuracy and deflate IP for young selection candidates. As error rates increased, correlations with benchmark GEBV declined and regression coefficients moved further from their ideal values, indicating reduced precision. Although error-free genotypes remain the clear standard, our findings show that an error rate of approximately 5% still yields acceptable levels of bias and dispersion, supporting reliable estimation of genetic gain and appropriate shrinkage of IP-based GEBV (Tsuruta et al., 2021). These results underscore the need for high-quality genotypes, especially for animals whose evaluations rely on IP, and highlight the substantial benefits of rigorous quality control in modern GS programs.
